# Traces Of Laboratory Earthquake Nucleation In The Spectrum Of Ambient Noise

**DOI:** 10.1038/s41598-018-28976-9

**Published:** 2018-07-17

**Authors:** Gevorg G. Kocharyan, Alexey A. Ostapchuk, Dmitry V. Pavlov

**Affiliations:** 1 Institute of Geosphere Dymanics of Russian Academy of Sciences, 119334 Moscow, Russia; 2 Moscow Institute of Physics and Technology, 141700, Dolgoprudny, Moscow Region Russia

## Abstract

The short-term forecast of earthquakes associated with fault rupture is a challenge in seismology and rock mechanics. The evolution of mechanical characteristics of a local fault segment may be encoded in the ambient noise, thus, converting the ambient noise to an efficient source of information about the fault stress-strain conditions. In laboratory experiments we investigate micro-vibrations of a block-fault system induced by weak external disturbances with the purpose of getting reliable evidence of how the system transits to the metastable state. We show that precursory changes of spectral characteristics of micro-vibrations are observed for the complete spectrum of failure modes. In the course of experiments we systematically change the properties of interface to perform the transition from stick-slip to steady sliding and observe the characteristics of micro-vibrations of the laboratory block-fault system. Detected were systematical alterations of the system natural frequency and those alterations were determined by the evolution of fault stiffness. The detected regularities suggest that the final stage of seismic event preparation can be revealed in analyzing the spectral characteristics of ambient noise. The detection of natural oscillations of a block-fault system can be a new useful tool to monitor active faults in real time.

## Introduction

The concept that frictional instability is the most likely mechanism of shallow earthquakes is now dominating in seismology and rock mechanics^1,2^. Taking laboratory stick-slip as a small-scale mechanical representation of the processes in the seismic source, various aspects of seismic source phenomena were studied in detail such as friction law, radiation efficiency, precursory effects, etc^3–7^. Such tests are by no means a sort of scale modeling since it is simply impossible to fulfill all the similarity criteria in this case^8^. They should be considered more as a study of fundamental properties of geomaterials and their structural properties which determine fault evolution.

One of the problems being solved in seismology is the search for macroscopic characteristics that could be used in monitoring seismically active areas in order to predict earthquakes. The phenomena of seismic quiescence^9^ and foreshock activity^10–12^, variations of geo-acoustic emission^13^ and water level in wells^14^, radon anomaly^15^, alterations of velocities of seismic wave propagation^16–18^, Q-factor of the medium^19^, physical and chemical properties of fluids^20^, and others were considered as earthquake precursors. Some analogues of the above phenomena are regularly observed in laboratory experiments^7,21–24^. The listed phenomena do not characterize the rupture zone itself, they rather deal with a larger area of elastic energy accumulation. For this reason, considerable efforts have not resulted so far in any reliable short-term earthquake precursor, although some positive progress has been made at least in post mortem precursory signal analysis^25^.

As an alternative to observing characteristics of a huge area where earthquake precursors may be revealed, various methods of local monitoring seismically active faults can be used. Seismic methods seem to be most suitable for this purpose since they are supported by excellent instrument provision, as well as by well developed techniques of processing the data on kinematic and dynamic characteristics of vibrations in different frequency bands^26,27^.

Below we present laboratory experiments on a spring-slider model set-up designed to study the possibility to recover some information about fault stress-strain conditions from the ambient seismic noise. To investigate the complete spectrum of frictional slip behaviors, we altered gouge content in the model fault. The ambient noise was imitated by slider micro-vibrations produced by the acoustic white noise of a loudspeaker. In a greater detail the description of experiments is presented in the section Methods. We investigate spectral characteristics of ambient noise in detail and detect the main resonant frequencies. We find some reliable evidences of a laboratory earthquake nucleation associated with the evolution of mechanical characteristics of the fault.

It is well known that damaged rocks (for example, in a fault core) typically have a nonlinear behavior^28–30^. The effect of non-linearity becomes particularly striking when the acting stresses are close to the fault ultimate strength. In particular, the non-linear deformational behavior can explain the effects we observe in experiments presented below.

We find it convenient to characterize the deformation properties of a fault by its normal and shear stiffnesses^31^:

1 $${\kappa }=\frac{\partial \sigma }{\partial x},k=\frac{\partial {\tau }}{\partial u}$$

where *σ* and *τ* are the normal and shear effective stresses acting in the vicinity of discontinuity, *x* and *u* are the relative normal and shear displacements of its sides. Differentiating the stress-displacement curve *τ*(*u*) allows us to measure the static value of fault shear stiffness. Fault slip modes are controlled by the ratio of two parameters – the maximal rate of frictional resistance weakening of the fault (the specific unloading stiffness of the fault) to the stiffness of enclosing rock massif^4,6^. The transition from stick-slip to aceismic creep has been studied in detail both analytically and in laboratory experiments^2,6^.

As shear stresses approach the limit of frictional strength, and the source zone transits to the metastable state, the shear stiffness of the source zone should noticeably decrease^29,32^. At the final stage of shear stress growth the stiffness can become very small, however, the dynamic rupture accompanied by elastic wave generation starts only when the modulus of the shear resistance decrease (or, in other words, the stiffness of the fault at the unloading branch) becomes larger than the stiffness of the surrounding rock. Though the static stiffness of a fault cannot be measured *in situ*, laboratory experiments demonstrate correlation between the static and dynamic fault stiffnesses^33^. The normal and shear dynamic stiffnesses of a fault can be estimated through measured parameters of seismic waves transmitted through it. The theory of seismic waves interacting with a cut in an elastic medium is developed rather exhaustively^34,35^. We should take into account the strong non-linear dependence of the dynamic stiffness (estimated by seismic methods) on the intensity of disturbance^28^. Ultrasonic precursors are observed in laboratory experiments during stick-slip just before the failure of the frictional discontinuity occurs. Those precursors are attributable to a decrease of the shear stiffness of discontinuity^36,37^. Precursory changes of seismic velocities occur in laboratory faults for a full range of failure modes similar to those of tectonic faults^7^.

It is likely that a slow preliminary sliding and, consequently, a decrease of fault stiffness before the dynamic rupture does exist in nature. According to the pre-slip model, prior to a large earthquake, an area emerges around the hypocenter, where the pre-slip occurs. Some authors assume, that the size of this pre-slip area is proportional to the final earthquake magnitude^11,38–42^. However, it should be emphasized that many authors concern in pre-slip model. They observed irregular patterns observed at the final stage of an earthquake nucleation^43,44^.

It is clear, that stress and deformation monitoring *in situ* with the purpose to forecast the moment of earthquake is in fact impossible so far, because of insoluble difficulties associated with regular measurements of stresses and displacements at seismogenic depths. The seismic method of fault stiffness monitoring seems more suitable *in situ*. But in practice, it is difficult to perform active monitoring of seismogenic faults. To control the stiffness of a local fault section, first of all, it is necessary to choose the section itself, which is, naturally, very problematic. Besides, it is desirable to have records of both the incident and the transmitted waves, which is often impossible. In this regard, using the ambient noise to control the conditions of a seismogenic fault seems to be an exciting idea^45,46^.

The spectrum of ambient noise should inevitably contain components corresponding to natural frequencies of a blocky structure. One set of frequencies – the natural frequencies of elastic blocks – origins directly from the reflections of waves from interblock boundaries (just like standing waves in a resonator) and is defined by the characteristic block size:

2 $${{f}_{bl}}^{i}=\frac{c}{2L}$$

where *C* is the velocity of propagation of longitudinal or shear wave and *L* is the characteristic block size, which is determined by the structure of a rock massif and the frequency of seismic waves used for investigations.

The other set of frequencies originates in a blocky medium, because some blocks, separated one from another by rather compliant faults, can execute free harmonic oscillations triggered by external disturbances^47^. If the Q-factor of such a block-fault system is not extremely low, the natural frequency of these free harmonic block oscillations should be detectable in the spectra of recorded ambient seismic noise^47^.

The simplest 1D mechanical analogue of such an oscillating system is the harmonic “mass on spring” oscillator (inset in Supplementary Fig. S1). Then, the natural frequency of the oscillating block-fault system can be estimated as follows (Supplementary Section S1):

3 $$f=\frac{1}{2\pi }\sqrt{\frac{k}{\rho \cdot L}}$$

A preliminary estimation of specific frequencies of this set can be performed according to the empirical relation derived from seismic measurements of the dynamic (estimated by seismic methods) stiffness *k* of fractures and faults of different lengths *L*^28,48,49^:

4 $$k\approx {L}^{-0.32}GPa/m$$

So, for blocks tens of kilometers in length, the characteristic periods of oscillations can reach tens of seconds. If a fault approaches the metastable state, and its shear stiffness decreases, the specific natural frequency that could be detected in the spectrum of ambient seismic noise should decrease too.

**Figure 1:**
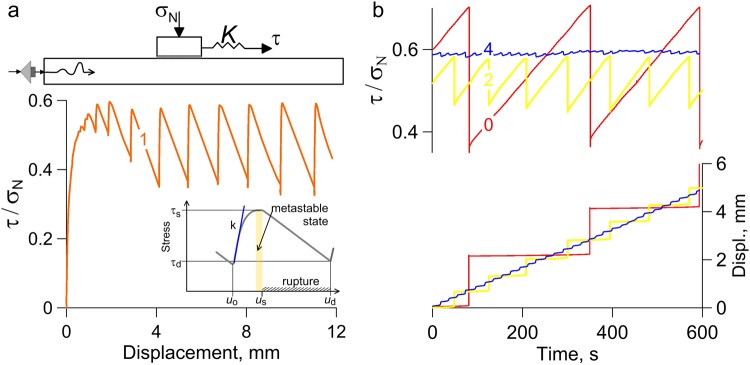
(**a**) Sketch of the experimental set-up and variation of friction as a function of displacement in experiment no. 7 (Suppl. Table S1). During an experiment the granite block slides along the surface of the granite rod covered with a layer of granular material. The sliding block is loaded with the normal (σ_N_) and shear (τ) loads plus periodic external acoustic vibrations. The stiffness of shear loading system is constant and equals to *K* = 55 N/mm. The inset shows schematically the stress diagram of a laboratory “seismic cycle”. At the loading stage, the fault state can be described using the fault shear stiffness *k* (static value). As the shear stress approaches the fault frictional strength (τ_s_), the fault transits to the metastable state and its stiffness *k* decreases radically (down to zero). (**b**) Friction data for experiments on fault interfaces with varying fillers (quartz sand/clay gouge with the clay content: 0–0%, 1–1%, 2–2%, 4–4%).

The reality of the above scenario was verified in laboratory tests at a slider set-up, where a block under normal and shear loads slides along a base, while weak stochastic vibrations are excited in the base by a special striker (see Methods). The fault model was filled with a layer of granular material composed of quartz sand and clay. First, we observe a spectrum of slip behaviors as a function of quartz sand/clay gouge composition (Fig. 1 and Supplementary Fig. S4). We systematically vary clay content in the gouge and document a transition from fast, dynamic stick-slip, accompanied by audible energy radiation, to silent slow-slip events^6,50^.

**Figure 2:**
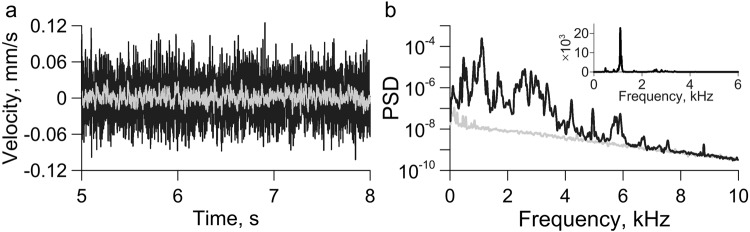
Examples of records of block velocity (**a**), and their spectra (**b**) under the striker action (black) and without it (gray). The inset shows the ratio of spectral amplitudes.

Weak acoustic pulses in the base produce micro-shear-deformation of the interface, which, in turn, excites oscillations of the upper block relatively the base (Fig. 2). In a preliminary experimental series, we verified that the oscillations being excited have no effect on the macroscopic parameters of slip events – peak velocity, recurrent time, fault strength. Natural harmonic oscillations of the moveable block that are controlled by the shear stiffness of the interface can be detected in the spectra of recorded signals. The spectra evidently contain the main maximum in the frequency range of 900–1100 Hz, as well as the maximum of a lower amplitude in the frequency range of 500–600 Hz (Fig. 2b). These specific maxima manifested in all experimental series including the complete collection of slip modes. The frequencies of natural oscillations were estimated using the spectral centroid^51^, which in the frequency range of (f_1_, f_2_) was calculated as follows:

5 $$fc=\frac{{\sum }_{{f}_{1}}^{{f}_{2}}{f}_{i}{A}_{i}}{{\sum }_{{f}_{1}}^{{f}_{2}}{A}_{i}}$$

Hereinafter the first main maximum corresponds to the extended frequency band of 750–1250 Hz, the second maximum – to the frequency band of 400–650 Hz (Fig. 2b, inset).

**Figure 3:**
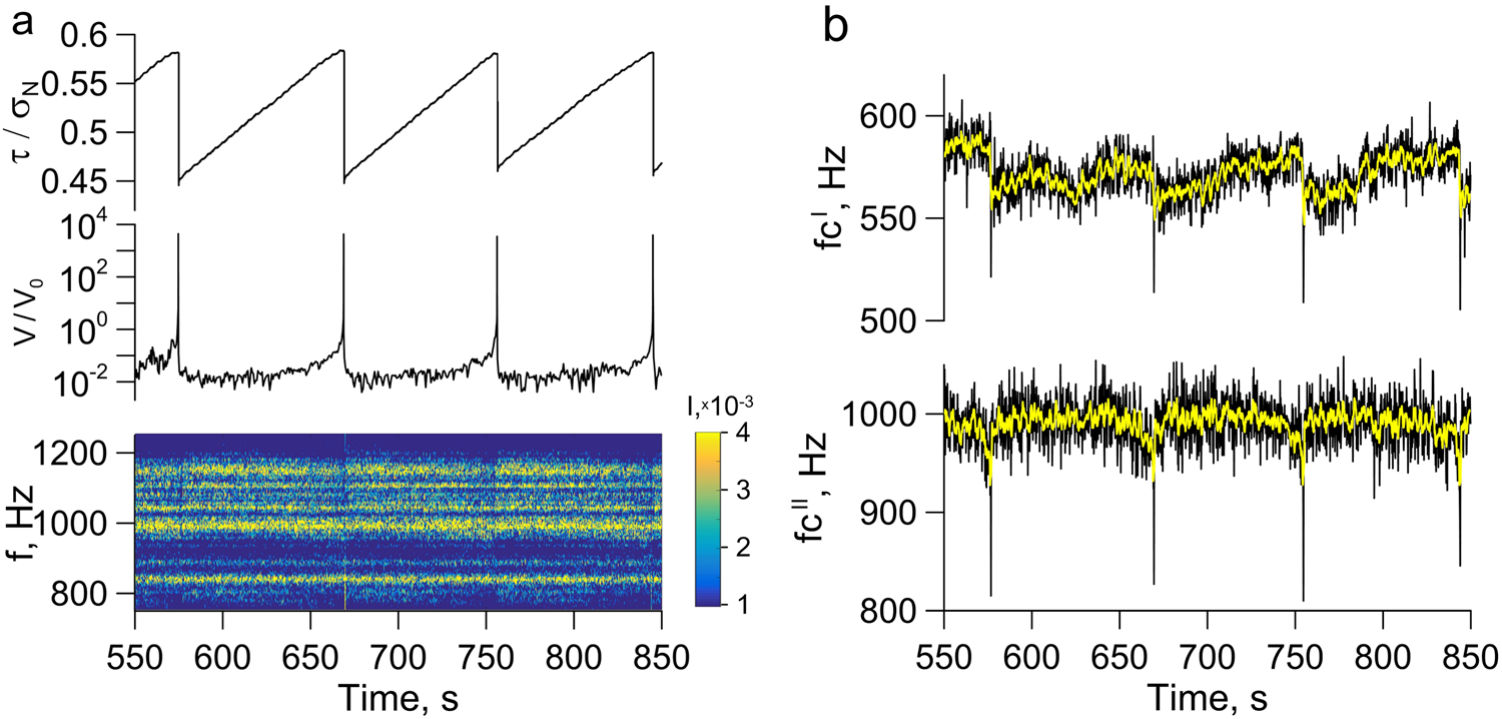
(**a**) Friction and velocity of sliding scaled by the velocity of loading V_0_ versus time, and the spectrogram of block vibrations in the frequency band of 750–1250 Hz in the experiment no. 3 (Supplementary Table S1). (**b**) Alterations of the spectral centroid with time in the frequency bands of 400–650 Hz and 750–1250 Hz. The yellow line is the moving average of the spectral centroid for the period of 1 s.

The combination of microseismic and mechanical characteristics provides a comprehensive view of the fault state evolution during a seismic cycle (Fig. 3, Supplementary Fig. S4). Repeating acts of dynamic instability may be considered as laboratory analogues of seismic cycles. After the dynamic rupture occurs, the gouge-filled interface strengthens gradually. Despite the shear stress increase, the slip velocity gradually decreases. Then the period of relative stability comes – the “interseismic” stage – the block moves at the minimal, approximately constant velocity. The shear stiffness reaches its maximal value of *k*_*o*_^52^_._ Duration of this stage essentially depends on the type of events being realized: the more intensive the event is, the longer the “interseismic” stage is. At the final preseismic stage the accumulating elastic energy and deformation results in initially slow, and just before the rupture – in a dramatic increase of slip velocity.

In the frequency range of *Δf*^*I*^ (400, 650) Hz dynamic slip episodes are accompanied by an abrupt decrease of the spectral centroid *fc*^*I*^. However, changes of the value of centroid at the “interseismic” stage do not correlate with alterations of stress-strain conditions. In the range of *Δf*^*II*^ (750, 1250) Hz alteration of the value of spectral centroid coheres with the stages of slip mode. Sections that correspond to the four stages of slip cycle can be clearly traced in the range of *Δf*^*II*^. During the dynamic rupture, the value of *fc*^*II*^ comes to minimum. After the rupture stops, *fc*^*II*^ grows fast reaching its maximal value of $f{c}_{0}^{II}$, which corresponds to the characteristic value of shear stiffness *k*_*o*_ at the loading stage. At the “interseismic” stage variations of the spectral centroid *fc*^*II*^ are relatively small. During the process of elastic energy accumulation, long before the ultimate strength is reached, *fc*^*II*^ starts to decrease, the rate of decrease gradually growing as the moment of dynamic rupture approaches. We can assume that it is the frequency range of *Δf*^*II*^ that corresponds to the main tone of natural harmonic oscillations of the laboratory block-fault system and carries the information about evolution of the block-fault system and its transition to the metastable state. The fact that the main natural frequency of the system lies in the frequency range of *Δf*^*II*^ is supported by independent special experiments at a vibration exciter (see Supplementary Section S2 for details). It is worth mentioning that using vibrations of higher amplitudes leads to a more pronounced effect of *fc*^*II*^ alteration (Supplementary Fig. S5).

**Figure 4:**
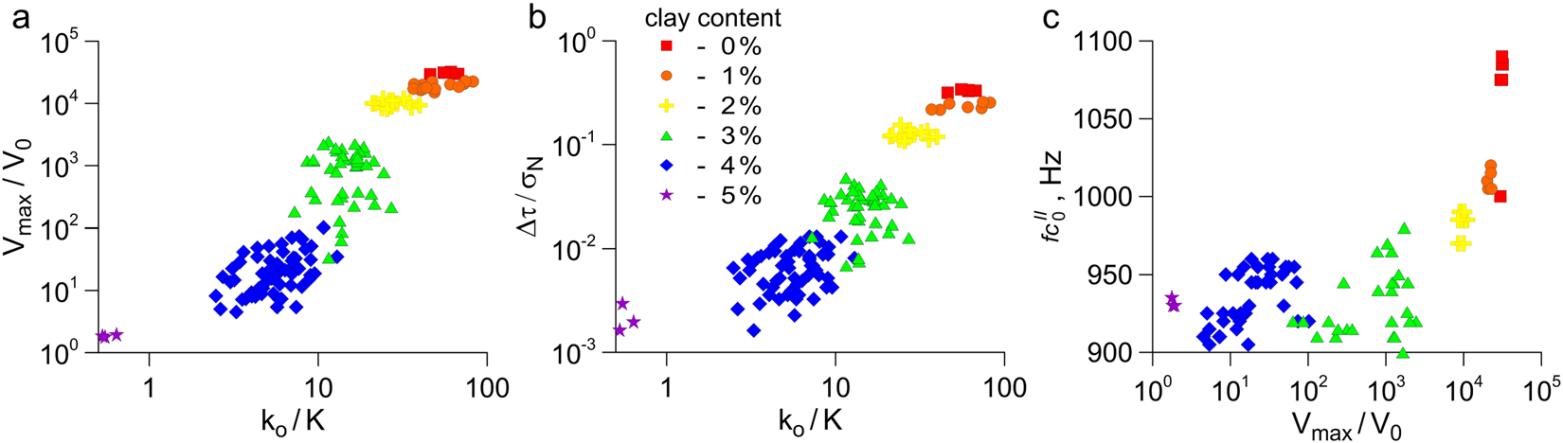
Variations of peak velocity (**a**) and stress drop (**b**) as functions of the ratio of the interface shear stiffness *k*_*o*_ to the stiffness of loading system K. (**c**) Correlation of the peak velocity with the natural frequency of fault-block system $f{c}_{0}^{II}$.

The above mentioned regularities of the spectral centroid *fc*^*II*^ behavior during the laboratory seismic cycle are typical for all slip modes, from dynamic failures to slow slip events. Decreasing the intensity of dynamic events is accompanied by a decrease of the spectral centroid $f{c}_{0}^{II}$ value (Fig. 4). Decreasing the intensity of dynamic events leads also to a decrease of the amplitude of variations of the spectral centroid at the “pre-seismic” stage: for the “fastest” events the value of spectral centroid decreases by 75–80 Hz, while for “slow” events the decrease is about 20–30 Hz (Supplementary Figure S3 and Table S1).

Our experiments show that seismic event nucleation is controlled by deformation characteristics of the local section of a fault. Slight variations of the interface structure, that have no noticeable effect on the fault strength, can result in a radical change of the fault slip mode (Fig. 4). Our results support the idea, suggested earlier, that the major factors dictating the mode of fault slip are the frictional properties and the characteristic value of shear stiffness of the fault zone^6,50,52^. The seismogenic rupture, as a rule, nucleates at the fault section exhibiting an unstable sliding, while the sections that hamper and stop the rupture can be either stable or conditionally stable ones^2,53^. Probably, the higher the fault stiffness is, the higher the frequency of natural oscillations of the block-fault system should be.

Although fault sizes and stress conditions in natural and laboratory studies differ, and a more comprehensive discussion is required, the similarities of the evolution of mechanical characteristics noticed in natural observations and in our tests may suggest applicability of our findings to natural faults. Judging by the obtained results, under a continuous weak external disturbance, modes of free harmonic oscillations of a block-fault system can originate, and their frequencies are governed by the fault shear stiffness in accordance with equations (3) and (4).

Analysis of the ambient seismic noise, containing traces of those oscillations, can provide an important information about stress-strain conditions of the fault and its transition to the metastable state. It should be emphasized that it is unclear so far, what characteristic size should be used *in situ* for estimations by equations (3) and (4). This size may vary from the size of nucleation zone *L*_*c*_^2^ to the size of the order of the length of future fault rupture, as it is in the laboratory experiment. If we take $L \sim L{}_{c} \sim {10}^{3}\,{\rm{m}}$, the frequency of natural oscillations of the block-fault system will be of about 5 Hz. If we assume the characteristic size to be equal to the rupture of a large earthquake $L \sim {10}^{5}\,{\rm{m}}$, the frequency of natural oscillations at the interseismic stage should be of about 50 mHz, and in transition to the metastable state the frequency will decrease essentially. While it is doubtful to record natural oscillations of a block-fault system in the high-frequency band of *f* > 0.1 Hz due to wave attenuation with distance and due to high noise level of secondary microseisms, these oscillations could be recorded in the low-frequency band^54,55^. Concerning the mechanism of excitation of natural oscillations of such large-scale objects as blocks of the Earth’s crust tens of kilometers in size, one should, evidently, suggest that in order to initiate such a process, bulk forces with non-zero gradient at distances of about of block length (tens of kilometers) are required. A possible trigger of such oscillations can be, for example, surface waves of distant powerful earthquakes.

It is worth mentioning that our experimental results are supported by a similar pattern of alteration of the characteristic frequency of oscillations, which was observed before several earthquakes^54^. The most vivid manifestation of this phenomenon happened on the eve of the M9.2 Sumatra earthquake, 26 December 2004, where the period of such oscillations (the oscillations appeared more than two days before the main shock) had been increasing rapidly as the moment of the main event approached (See Fig. 17 in ref.^54^). It is important to note that those oscillations were triggered by seismic waves from another distant earthquake near the island of Macquarie (23 December 2004, M=7.9) that took place about 7000 km away. In other cases (М7.8 Kronotskoe earthquake, 5 December 1997; M8.3 Hokkaido earthquake, 25 September 2003) triggering effects of external disturbances were also reported: very low frequency oscillations have been detected synchronously at several stations^54^. Low frequency oscillations with periods of up to 200 s were also observed some time before the main shock of the event that occurred in the North-West Pacific region^55^.

The process of fault recovering after an earthquake was traced in a number of works using trapped waves^56,57^.

Previous experiments^7,36,37^ have revealed the change in velocity speed during stick-slip instabilities. For such an effect to be detected *in situ*, one must have both the source of seismic waves and the measuring profile intersecting the fault zone. Our experiments have demonstrated that the shift of the characteristic frequency of natural oscillations toward low frequencies may serve as an indicator of a dramatic decrease of the fault shear stiffness and the transition of the block-fault system to a metastable state. It is evident that detecting natural frequencies of blocks in the ambient noise is undoubtedly a separate problem, requiring special methods of observations and data processing. Apparently, most advantageous for detecting the characteristic values for a certain region, can be the intervals of seismic records after the passage of surface waves from distant earthquakes. These vibrations with periods of several tens of seconds have noticeable amplitudes and rather long durations, which promotes excitation of resonant harmonic oscillations of block-fault systems. Determination of such frequencies, specific for each area, and tracing their variations can, in our opinion, make the base for a new approach to local monitoring of seismogenic faults and to solution of the problem of short-term earthquake forecast.

## Methods

All the experiments were performed in the classical statement of the slider model (Supplementary Fig. S1). The granite block 8 × 8 × 3.2 cm in size and of the mass of 550 g was laid at the surface of the granite rod 2.5 m long and 10 × 10 cm in cross section, in the middle of it. The contact between rough surfaces of the block and the rod was filled with a layer of granular material 3 mm thick. The amplitude of surface roughness of the block and the rod was 0.5–0.6 mm. The gouge layer was prepared using the leveling frame, so that the initial thickness of the layer was the same in all the experiments. The filler was composed of the mixture of quartz sand (grain size of 200–315 µm) and clay (grain size 10–60 µm). Using dry quartz sand did not allow us to realize stick-slip under the normal loads applied. So, the filler was moistened with glycerol, 5% of the entire filler mass. Using glycerol allowed us to ensure test reproducibility, unlike water. Using water as the moistening fluid led to a strong scattering of parameters – mixture humidity changed noticeably in the course of test preparation and conduction. The maximal change of layer thickness to the end of an experiment did not exceed 1 mm. All experiments were performed at room temperature and humidity.

A constant normal static load was applied to the block through a special device that excluded origination of shear forces at the upper side of the block. The value of normal static stress was σ_N_ = 50 kPa. The shear force was applied to the block through a spring with the stiffness of 55 N/mm, which was pulled by the edge at the constant velocity of V_0_ = 8 µm/s. The above parameters remained the same in all experiments. The shear force was measured by a force sensor with the accuracy of 1 N. The block displacement relative to the base was measured by a laser sensor in the frequency range of 0–5 kHz with the accuracy of 0.1 µm.

For a detailed analysis, we chose the section of stress-deformation diagram, where the strength of the model fault reached its residual value of τ_s_ and the deformation events repeated quasi-periodically. Vibrations in the rod (the base of the set-up) were excited by impacts over its edge. The impacts were produced by a plane rough striker attached to the diaphragm of the coil driven loudspeaker, which was fed by the signal from the white noise generator. Elastic vibrations were measured with accelerometers Bruel & Kjaer 4344 mounted on the rod and on the block (no. 6 in Supplementary Fig. S1). These vibrations had no visible effect on the macroscopic parameters of slip regime - peak velocity, recurrent time, fault strength.

To investigate the complete spectrum of the frictional slip behaviors, we altered the gouge content in the model fault. We chose granular quartz and clay because they are common minerals in natural fault gouges and are well-studied materials with reproducible friction behavior. In the experiments, we controlled only those mechanical and kinematic parameters of the system that could be measured *in situ*.

Despite the low level of normal stresses and temperatures we believe that it is possible to draw an analogy to the nature, based on the similarity of rheological diagrams of laboratory and natural faults. The stick-slip mode corresponds to normal earthquakes under field conditions and the stable sliding corresponds to the aseismic creep. The tests clearly identified transient deformation events with low displacement amplitudes and very low slip velocities. The intermediate deformation modes are the analogues of such natural phenomena as low-frequency earthquakes, very low-frequency earthquakes and slow slip events^58^.

### Data availability

All the data that support findings of this work were collected on geomechanical test bench of the Institute of Geosphere Dynamics of Russian Academy of Sciences. All the data presented in this study are available from corresponding author on request.

## Electronic supplementary material

Supplementary materials
