# Supplementary material for: Traces Of Laboratory Earthquake Nucleation In The Spectrum Of Ambient Noise
Source: Sci Rep. 2018 Jul 17;8:10764. doi: 10.1038/s41598-018-28976-9 (PMC6050260; doi:10.1038/s41598-018-28976-9)
Supplement: Supplementary file 1 — Supplementary materials [file 41598_2018_28976_MOESM1_ESM.doc]

**TRACES OF LABORATORY EARTHQUAKE NUCLEATION IN THE SPECTRUM OF AMBIENT NOISE**

Gevorg G. Kocharyan^1,2^, Alexey A. Ostapchuk^1,2^* & Dmitry V. Pavlov^1^

^1^Institute of Geosphere Dymanics of Russian Academy of Sciences, 119334, Moscow, Russia
^2^Moscow Institute of Physics and Technology, 141700, Dolgoprudny, Moscow Region, Russia
*Corresponding author: [ostapchuk@idg.chph.ras.ru](mailto:ostapchuk@idg.chph.ras.ru)

**Supplementary Materials**

**Supplementary Section S1. Natural frequency of a block-fault system**

Elastic vibrations in a blocky medium acquire specific spectral features reflecting both the blocky structure of the Earth's crust and the processes of stress accumulation and relaxation. One set of frequencies – the natural elastic frequencies of blocks – origins directly from the reflections of waves from interblock boundaries and is defined by the characteristic block size:

$f_{bl}^{i}=\frac{C}{2L_{i}}$, (S1)

where C is the propagation velocity of longitudinal or shear waves and L_i_ is the characteristic size of the block, which is determined by the structure of a rock massif and the frequency of seismic waves used for investigations.

The existence of another set of frequencies, was for the first time suggested by M.A.Sadovsky with co-authors^47^. They attracted attention to the fact that "...there exists a number of different-scale blocks, separated from "neighbors" by rather compliant layers (faults or fractures), and those blocks can perform free oscillations under external disturbances". If, the Q-factor of such a system is not too low, the natural frequencies of these "free" harmonic oscillations of blocks should be detectable in spectra of recorded ambient noise. The simplest 1D mechanical analogue of such an oscillating system is the harmonic oscillator mass M on a spring of the stiffness of K (Supplementary Figure S1). The corresponding natural frequency can be written as follows:

$f_{0}=\frac{1}{2\pi}\sqrt{\frac{K}{M}}$ (S2)

Let us use a simple scheme, where a block of the size of L×L×L oscillates on a gouge layer – a fault with normal and shear stiffnesses of  *κ* and *k*, respectively. Assuming that the layer stiffness does not depend on the amplitude of deformation (though, usually this assumption, is not true^28^, it can be used for estimations) one obtains the relationship between the layer stiffness and the effective stiffness of spring (K):

$K=k_{i}\cdot L^{2}$, (S3)

where *k_i_* is the corresponding values of normal  (*κ*) or shear (*k*) stiffnesses of the fault. Then, the natural frequency of the block-fault system is as follows:

$f_{0}=\frac{1}{2\pi}\sqrt{\frac{k\cdot L^{2}}{\rho\cdot L{}^{3}}}=\frac{1}{2\pi}\sqrt{\frac{k}{\rho\cdot L}}$ (S4)

**Supplementary Section S2. Amplitude-frequency response of a block-fault system.**

A special series of tests was performed at a Bruel & Kjaer (B&K) vibration exciter to demonstrate that the natural frequency of laboratory block-fault system lies in the range of *Δf^II^* (800-1200 Hz). The block of marble (3) 8×8×4.5 cm in size weighing 800 g with a rough base was put into a metal frame (2), which was attached by four screws to the B&K 4812 head mounted onto the B&K 4801 exciter (1). The whole assembly was oriented horizontally (Supplementary Figure S2). A piece of sandpaper (4) (grain size of 315-400 µm) was glued to the inner surface of the frame base. The 3 mm thick layer of quartz sand (5) moistened with glycerol (0.25% of sand mass) was put on the sandpaper. The block was pressed against the base by a calibrated spring (7) so that the normal force was equal to N = 315 N. The spring stiffness was K=55 N/mm, so the frequency of natural block oscillations related to the spring was 40 Hz (equation S2), which is almost two orders of magnitude lower than the band of interest. A thrust bearing (6) was placed between the spring and the block in order to exclude transmission of the tangential force. Vibrations of the system were measured with four accelerometers B&K 4344 (9). Two of them (horizontal and vertical) were glued to the lateral face of the block, and the other two (the same two components) - to the lower surface of the frame base. In addition, the standard accelerometer B&K 8305 (8) was mounted on the head to provide the feedback to the exciter. The operational mode was chosen so that the exciter maintained vibrations with the acceleration of constant amplitude at the head.

Recording the vibrations we got corresponding amplitude spectra, found the frequency response of the block-fault system and detected the main resonant frequencies.

During tests the exciter operated in the automatic sweep mode. Several experimental series were performed in slightly different frequency ranges and at different rates of frequency increase. The frequency changed in the ranges of 900-1300 Hz (2Hz/s) and 1000-1600 Hz (1Hz/s). Amplitudes of the "exciting" sweep vibrations were the same during all of the experiments. The tests were performed with the frame without the block, with the block lying directly on the sandpaper without the sand layer and, at last, with the block lying on the sand layer.

The comparison of these two experimental series has allowed us to identify the main natural frequency related specifically to vibrations of the block on the sand layer: $f_{0}=1140\pm20$ Hz (Supplementary Figure S3). This resonance is marked with the arrow.

Hence, in addition to necessary information, used in our main study, these experiments also supported the idea, that despite the low Q-factor of the system, the natural frequency of block vibrations on the layer in the direction parallel to the interface, may be detected in the spectrum of ambient seismic noise.

| 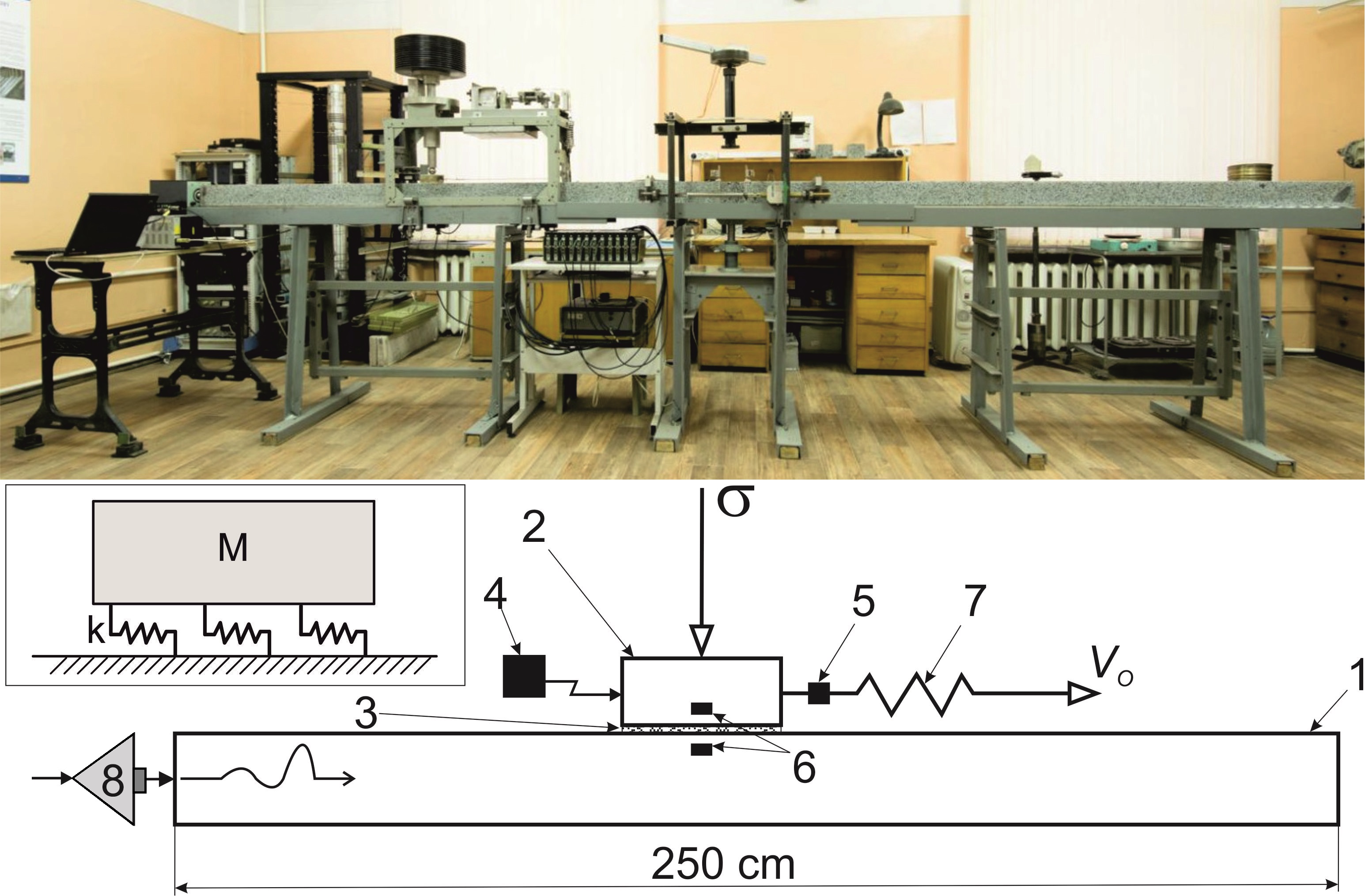 |
| --- |
| Supplementary Figure S1. The general view and scheme of experimental set-up.  (1) granite rod; (2) marble block; (3) filling layer; (4) displacement sensor; (5) force sensor; (6) accelerometers; (7) spring; (8) coildriven loudspeaker with rough striker fixed on the diaphragm.  The inset shows the mechanical 1D model of a block-fault harmonic oscillatory system. |

| 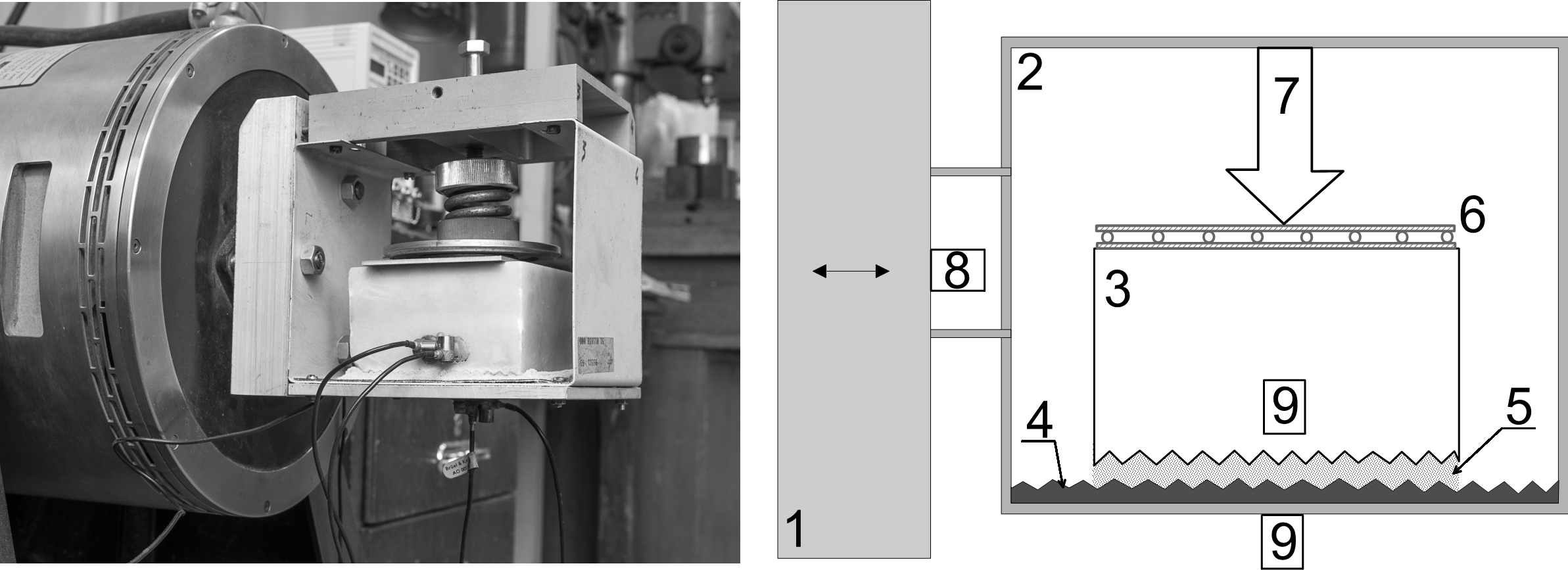 |
| --- |
| Supplementary Figure S2. The experimental set-up to measure the frequency response of the laboratory fault under uniaxial dynamic loading (left) and its scheme (right).  (1) vibration exciter; (2) metal frame; (3) marble block; (4) sandpaper; (5) layer of quartz sand; (6) thrust bearing; (7) calibrated spring; (8) accelerometer B&K 8305; (9) accelerometer B&K 4344. |

| 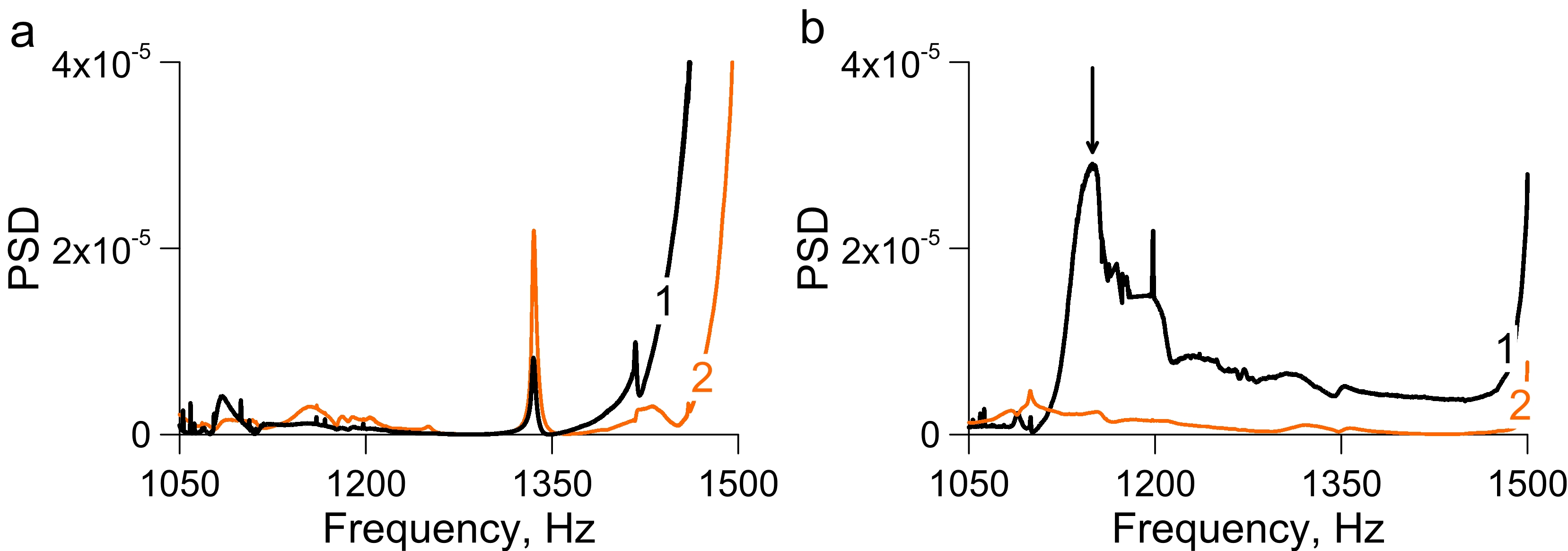 |
| --- |
| Supplementary Figure S3. Power spectrum density of vibrations of the block (1) and the frame (2), registered during the sweep 1000–1600 Hz at the rate of 1 Hz/s: (a) block without sand layer, (b) block on the layer of moistened quartz sand 3 mm thick. |

| 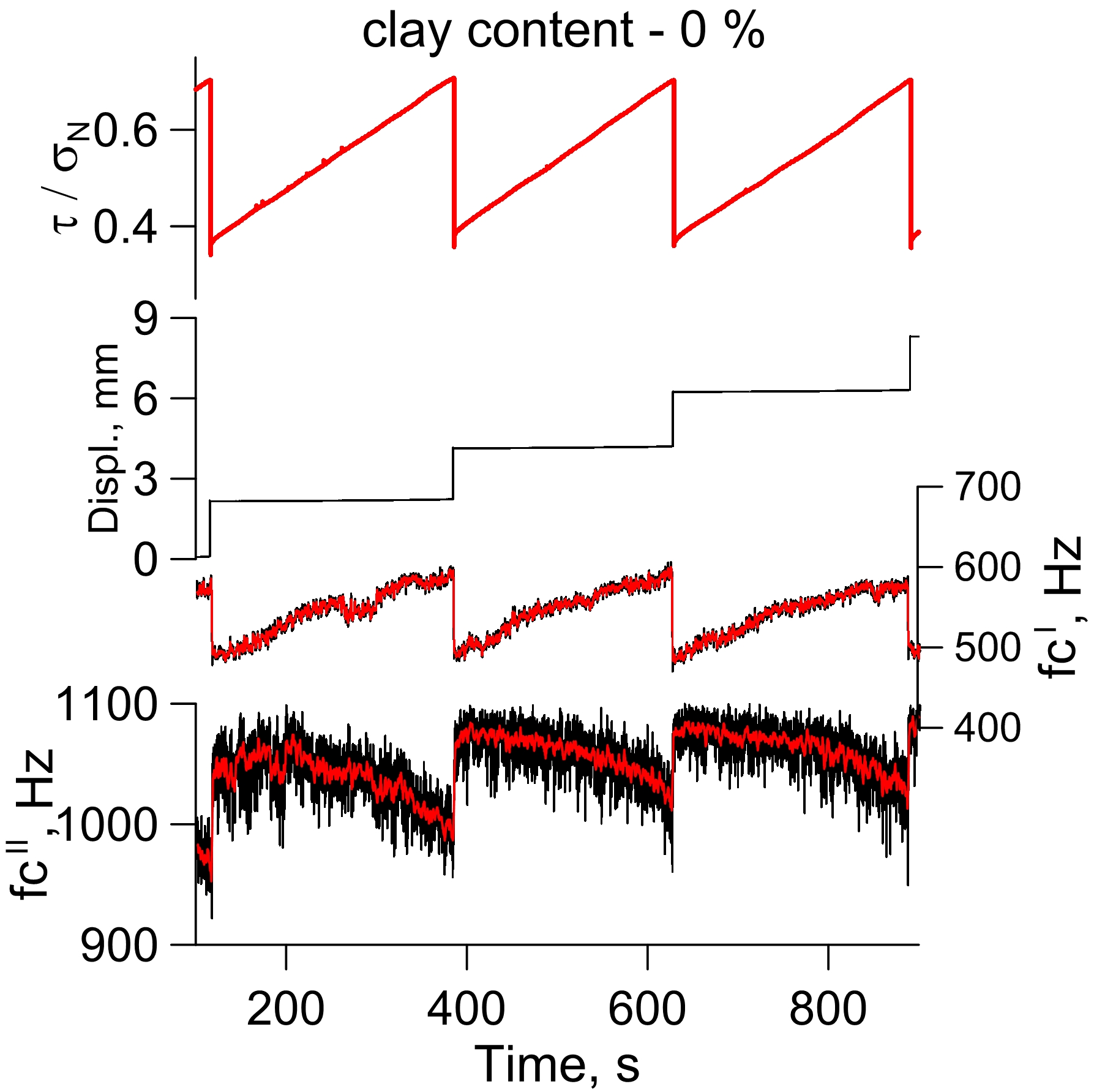 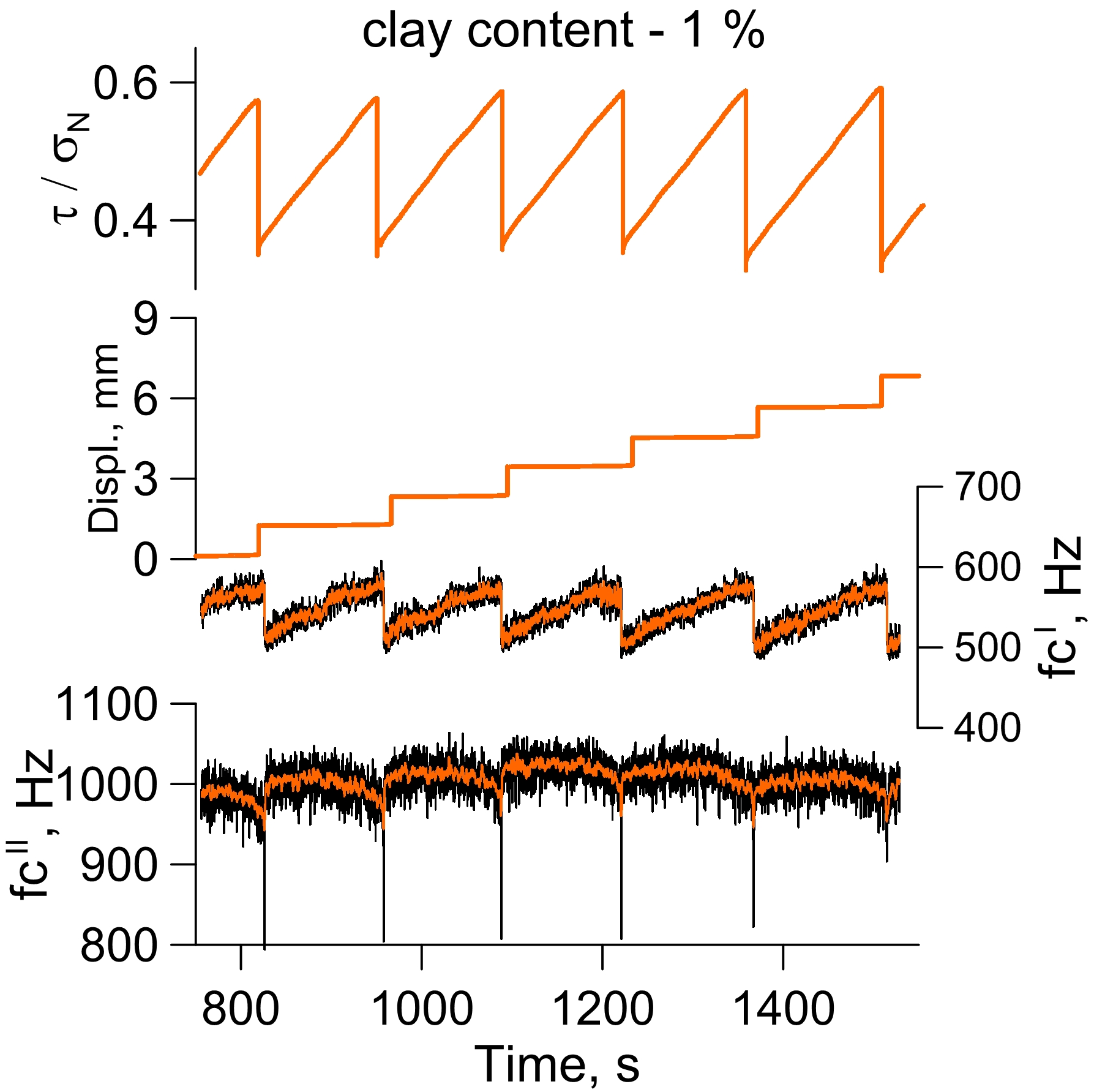  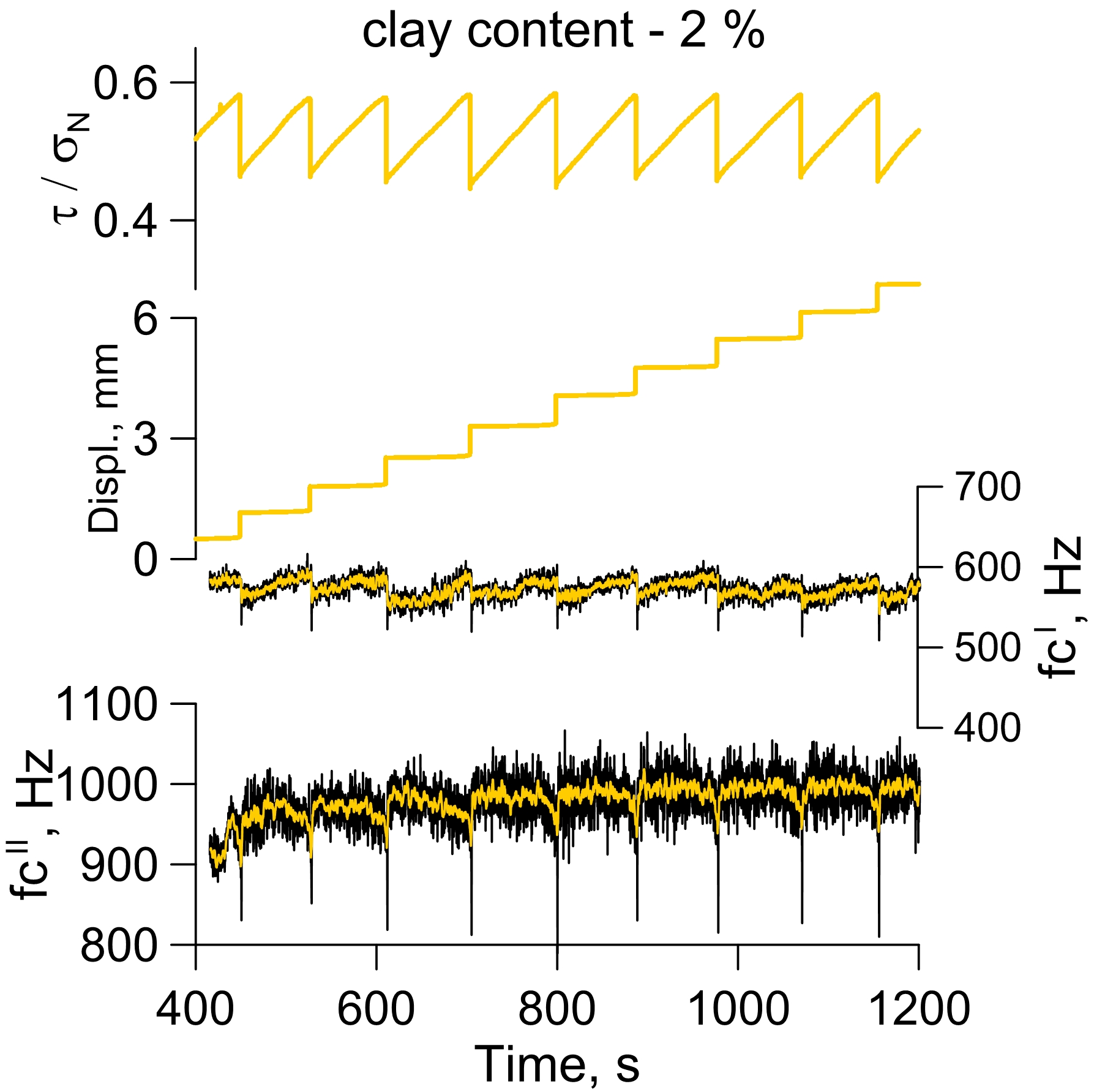 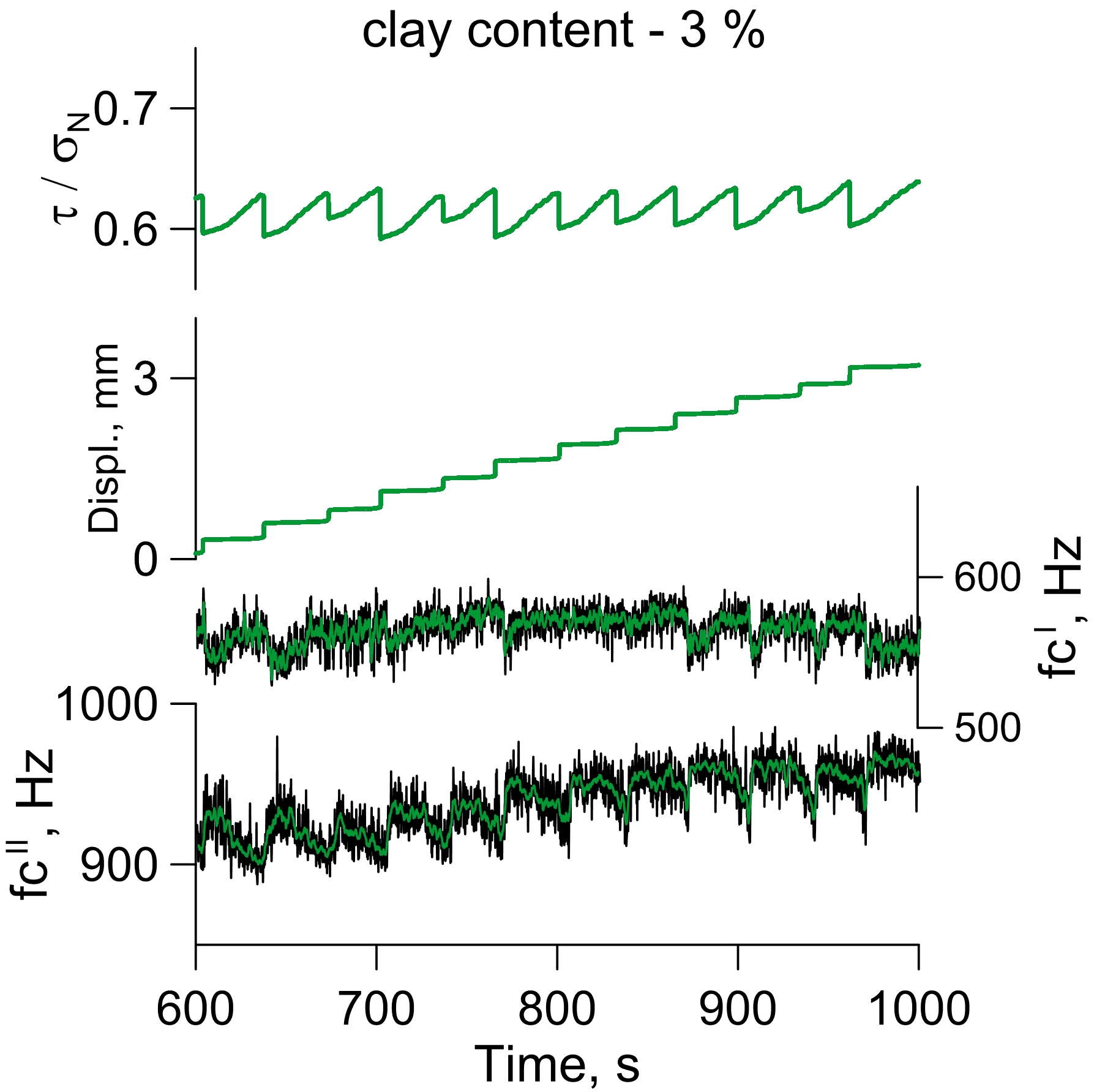  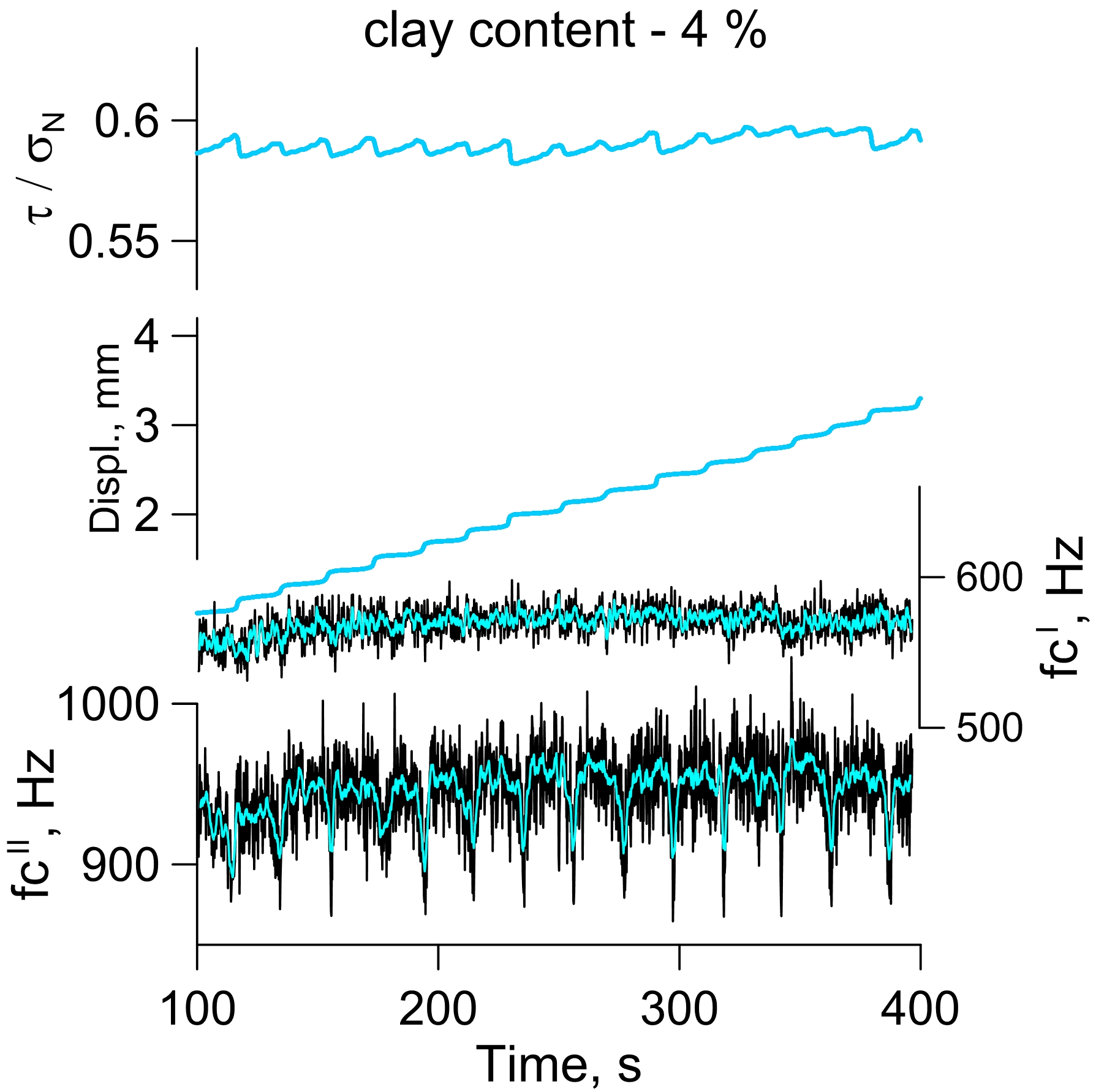 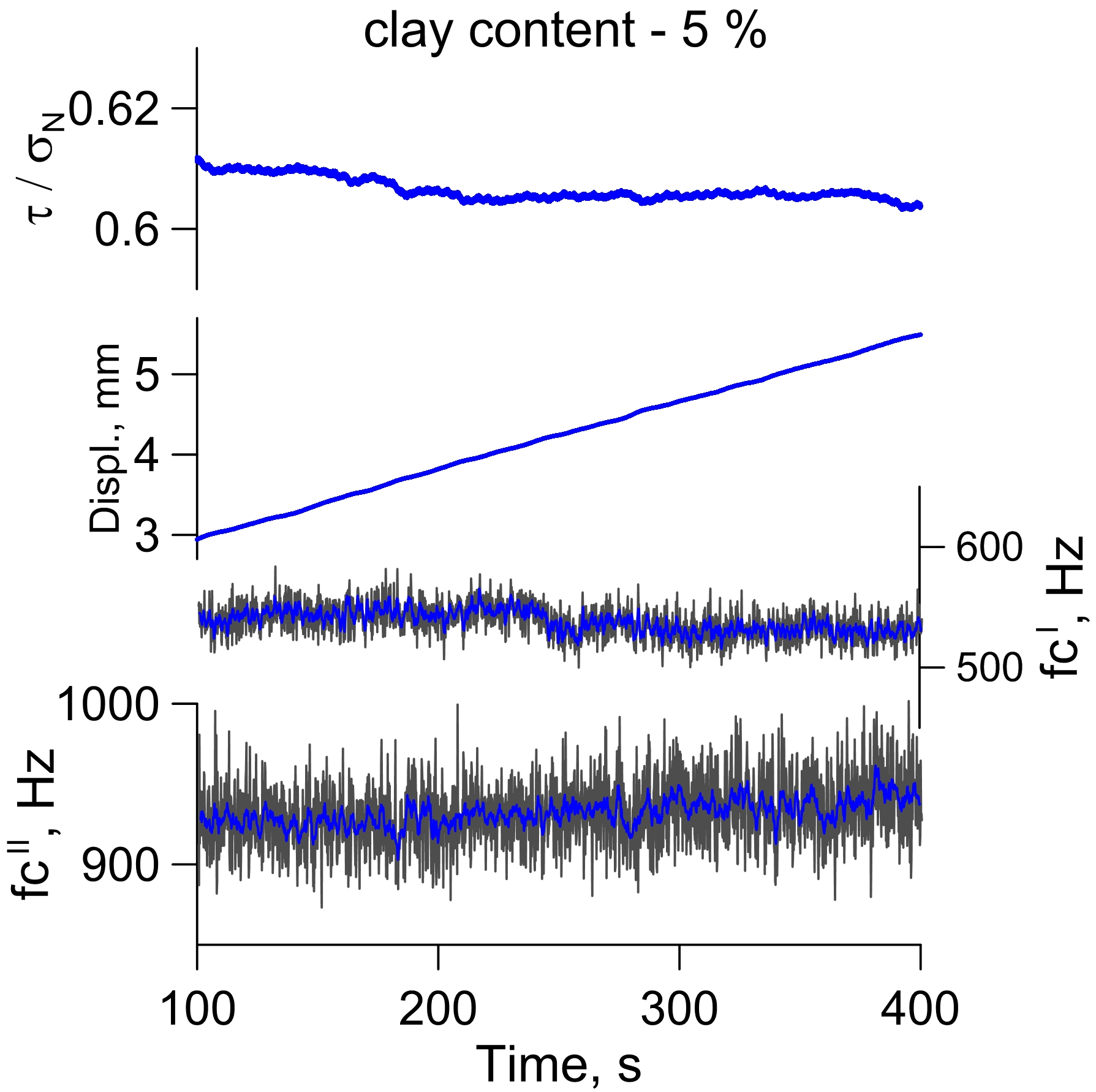 |
| --- |
| Supplementary Figure S4. Time variations of dynamic parameters of the system for different compositions of the gouge consisting of quartz sand and clay. The parameters under study are: normalized friction, block displacement and spectral centroid in the frequency bands of 400–650 Hz and 750–1250 Hz. |

| 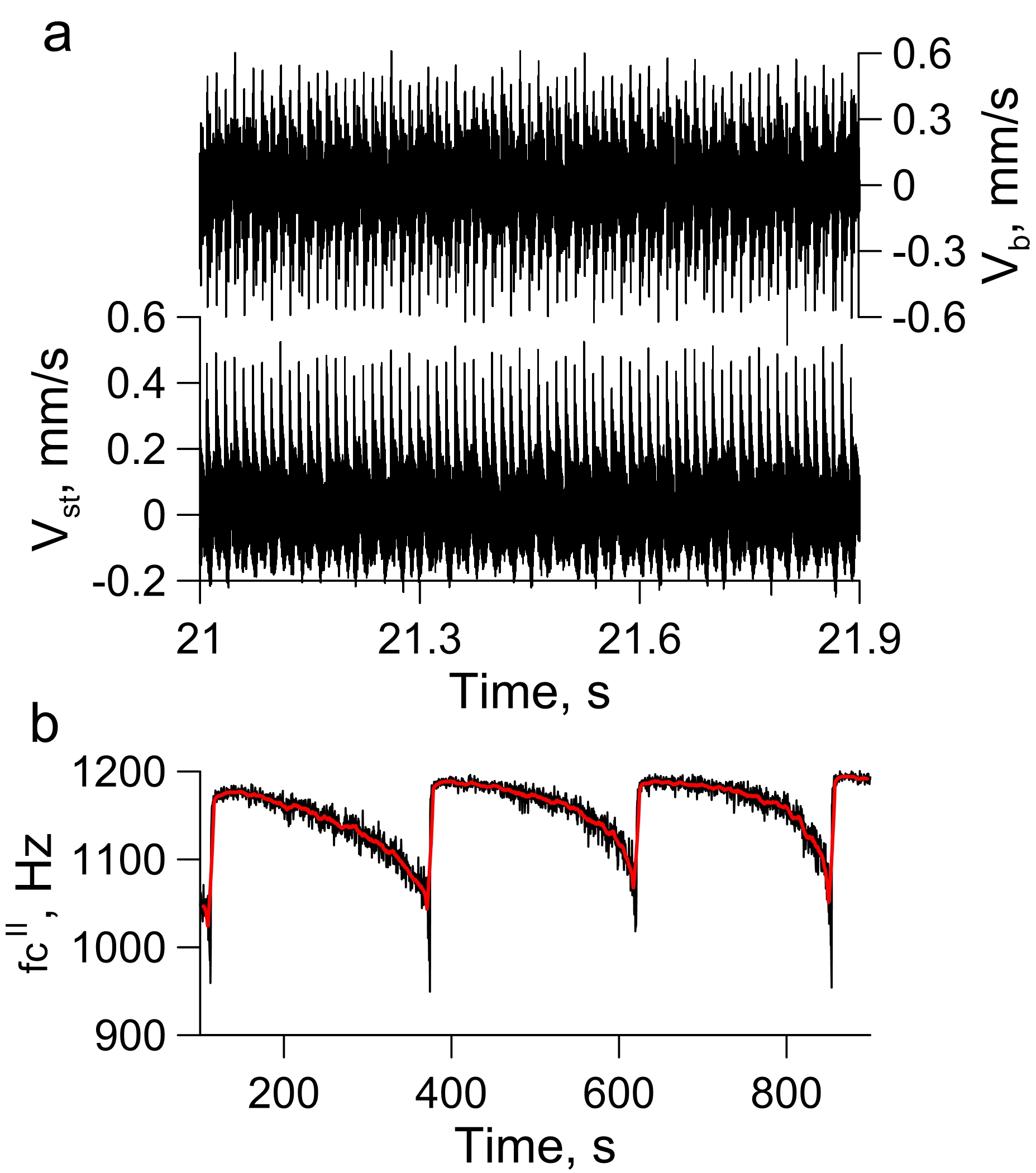 |
| --- |
| Supplementary Figure S5. (а) An example of high-amplitude disturbance (V_st_) propagating in the rod, and the reaction of the block (V_b_). The disturbance was produced by periodic impacts of a steel striker over the rod edge, and its amplitude exceeded the one of the main series by an order of the magnitude.  (b) Corresponding time variation of the spectral centroid for the interface filler composed of moistened quartz sand. |

| Experiment number | Clay content, % | V_0_, mm/s | Peak velocity V_max_, mm/s | Δτ / σ_N_ | natural frequency $fc_{0}^{II}$, Hz | $fc^{II}$variation at preseismic stage, Hz |
| --- | --- | --- | --- | --- | --- | --- |
| 1(27/0_1) | 0 | 0.022 | 210 ± 35 | 27 ± 4·10^-2^ | 1030 ± 20 | 50 ± 10 |
| 2(28/1_1,2) | 1 | 0.008 | 135 ± 8 | 19 ± 1·10^-2^ | 965 ± 20 | 50 ± 15 |
| 3(28/2_2) | 2 | 0.008 | 92 ± 8 | 13 ± 1·10^-2^ | 990 ± 5 | 55 ± 25 |
| 4(28/3_1,2) | 3 | 0.008 | 7.1 ± 5.6 | 23 ± 11·10^-3^ | 930 ± 10 | 20 ± 10 |
| 5(28/3_6) | 3 | 0.008 | 10.0 ± 4.8 | 29 ± 6·10^-3^ | 950 ± 20 | 25 ± 10 |
| 6(29/0_2) | 0 | 0.008 | 244 ± 16 | 33 ± 1·10^-2^ | 1080 ± 15 | 60 ± 10 |
| 7(29/1_1) | 1 | 0.008 | 159 ± 9 | 24 ± 2·10^-2^ | 1010 ± 10 | 50 ± 15 |
| 8(29/2_1) | 2 | 0.008 | 76 ± 7 | 12 ± 1·10^-2^ | 985 ± 10 | 45 ± 10 |
| 9(29/4_2) | 4 | 0.008 | 0.11 ± 0.05 | 5 ± 3·10^-3^ | 920 ± 10 | ~10 |
| 10(29/4_5) | 4 | 0.008 | 0.26 ± 0.15 | 8 ± 3·10^-3^ | 950 ± 10 | 15 ± 10 |
| 11(29/5_2) | 5 | 0.008 | 0.015 ± 0.001 | ~3·10^-3^ | ~930 | <5 |
| 12(29/35_2) | 3.5 | 0.008 | 1.07 ± 0.83 | 14 ± 4·10^-3^ | 985 ± 15 | 35 ± 15 |
| 13(23.03) | 10 | 0.008 | stable sliding | | | |
| 14(03.01/2) | 0 | 0.022 | 204 ± 20 | no record | 1135 ± 15 | 85 ± 5 |
| 15(26/1) | 5 | 0.022 | 0.055 ± 0.015 | no record | ~860 | – |

Supplementary Table S1. Summary of experiments. All test were conducted under the same environmental conditions.
